# Wear Analysis of NiTi Sand Screens Using Altair Discrete Element Method

**DOI:** 10.3390/ma17020281

**Published:** 2024-01-05

**Authors:** Azubuike Hope Amadi, Mysara Mohyaldinn, Abdullah Abduljabbar, Syahrir Ridha, Prasad Avilala, Gabriel Tayo Owolabi

**Affiliations:** 1 Petroleum Engineering Department, Universiti Teknologi PETRONAS, Seri Iskandar 32610, Malaysia; mysara.eissa@utp.edu.my (M.M.); abdullah_18002656@utp.edu.my (A.A.); 2 Institute of Hydrocarbon Recovery, Universiti Teknologi PETRONAS, Seri Iskandar 32610, Malaysia; syahrir.ridha@utp.edu.my; 3 Altair Engineering Inc., Kuala Lumpur 50470, Malaysia; prasada@altair.com; 4 Mechanical Engineering Department, Federal University of Technology Akure, Ondo 340110, Nigeria; owolabimee173392@futa.edu.ng

**Keywords:** NiTi, Oka model, Archard model, sand screens, wear, discrete element method

## Abstract

This research explores discrete element method analysis to investigate the wear of NiTi Sand Screens in comparison to traditional materials. The study utilized Altair EDEM v2022.2 software and employed Oka and Archard models to simulate the wear behavior of Nitinol, a well-established Shape Memory Alloy (SMA). The mechanical properties considered include Poisson’s ratio, solid density, shear modulus, and Young modulus. Results indicate significantly higher wear values and deformations with the Oka model compared to negligible wear with the Archard model. The Oka model’s emphasis on impact as the primary wear mechanism, supported by high normal cumulative energy, better represents sand screen wear phenomena. Additionally, this study indicates that factors such as particle size distribution and normal and tangential cumulative contact energy hold potential as predictors of wear response and characteristics. The Oka model demonstrated that NiTi exhibited reduced wear losses compared to SUS630 and Cr–Mn white cast iron, both of which are recognized for their high toughness when subjected to an impact load. Experimental analysis validated the simulation findings with morphological and graphical erosion plots. The limitation of observing the shape memory effect through DEM (discrete element method) simulation was acknowledged. Recommendations include characterizing post-wear microstructural changes, exploring the influence of temperature on wear behavior, and further research to refine wear models and understand SMA sand screen responses.

## 1. Introduction

Shape memory alloys are a class of smart materials that can undergo a reversible shape change when subjected to an external stimulus. These materials are widely used in various industries due to their unique properties such as excellent mechanical properties, high corrosion resistance, and biocompatibility. In the oil and gas industry, several materials are used as sand screens to prevent sand production during oil and gas extraction. However, sand screen wear can significantly affect their performance, resulting in reduced efficiency and increased operational costs. Therefore, this study suggests the use of Nitinol (NiTi), a popular shape memory alloy based on its mechanical properties, and that it is essential to analyze the wear behavior to improve their durability and reliability. 

The unique features of SMAs make them an excellent material choice for sand screens. SMAs are well known for their ability to restore their former shape following deformation, making them a good candidate for use in sand screens [1,2,3,4]. When exposed to mechanical stress or distortion, a sand screen can become damaged or deformed, reducing its efficacy in filtering sand particles [5]. Their ability to regain their previous shape even after deformation indicates that sand screens manufactured with SMAs may have a longer lifespan and be more effective at filtering sand particles. Furthermore, SMAs are known for their great strength and corrosion resistance, both of which are crucial features for materials used in sand screens [2,4,6]. SMAs are resistant to high temperatures and corrosive environments, allowing them to be used in difficult situations without eroding or deteriorating over time [7]. 

This study aims to analyze the wear behavior of Nitinol sand screens on both Oka and Archard models using the Altair EDEM simulator to suggest what model is more representative of the wear at the sandface of natural gas production wells.

### 1.1. Wear Control

Sand control is a critical issue in the oil and gas industry, and several techniques have been developed to prevent sand production during well completion and production. Sand screens made of different materials have been used to prevent sand particles from flowing into the wellbore. SMAs are an emerging class of materials due to their unique properties such as shape memory effect, superelasticity, and high corrosion resistance [2,8], and they possess properties that make them fit for wear control when used as screens. However, damage from wear, abrasion, and corrosion can seriously impair their performance, lowering efficiency and raising operating expenses [9,10,11].

Several researchers have studied the wear behavior of SMAs under different conditions. The Oka wear test is a popular method mostly used to evaluate the wear behavior of materials under indentation conditions. 

The wear behavior of NiTi-based SMAs under dry sliding conditions was examined and the results showed that the wear resistance of NiTi-based SMAs was affected by the sliding speed and load [12]. The wear resistance increased with increasing sliding speed and load. However, the wear resistance decreased with increasing sliding distance. The authors also found that the wear mechanism of NiTi-based SMAs was mainly due to adhesive wear and abrasive wear.

The wear behavior of SMAs under different conditions could be analyzed using wear models to predict the wear behavior for the purpose of wear control. Results from studies showed that the wear rate of SMAs is affected by the *normal load*, *impact angle*, *velocity*, and *hardness* of the material [7,13,14]. The authors also found that the wear mechanism of SMAs was mainly due to abrasive wear.

### 1.2. Wear Model

**Figure 1:**
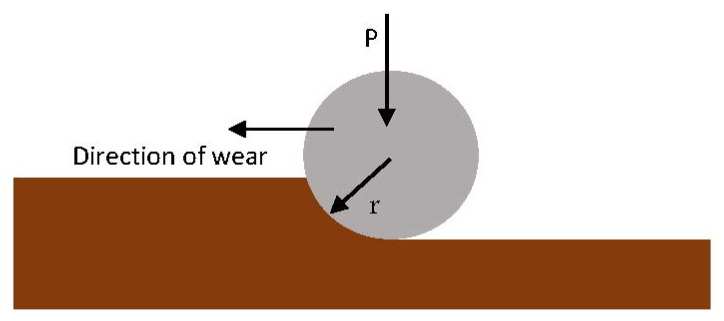
Surface wear design.

In describing the nature of material wear as shown in Figure 1, some parameters to consider are the *scratch hardness number* and *drag coefficient* because of the scratch [15]. The *scratch hardness number* ($H_{sc}$) can be defined as the resistance to surface penetration of a solid surface by moving a stylus with a tip radius while applying a constant speed and normal force. It is expressed mathematically as (ASTM: G171-03)

(1) $$H_{sc}=\frac{kP}{w^{2}}$$

where k is a geometrical constant, w is the scratch width, and P is the applied normal force. On the other hand, the drag coefficient D is the ratio of the scratching force ($F_{sc}$) to the applied force. It is expressed as

(2) $$D=\frac{F_{sc}}{P}$$

However, there are instances where the environment (air, moisture), precision, and bias can influence the nature of scratch hardness and drag [15]. In such cases, precautions should be taken to control the environmental parameter, record the relative humidity, and factor in tolerance for the bias.

There are some models that have been developed to estimate the wear of solid surfaces. The Archard wear model and the Oka wear model are established methods for assessing wear [16,17,18]. In certain applications, a relative wear model that integrates elements of both the Archard and Oka models is employed to provide a more comprehensive understanding of wear phenomena [18,19,20,21,22,23]. While the Archard wear, as proposed by Archard in 1953, was based on the assumption that the wear rate is proportional to the applied load and the sliding distance and is mostly used during machining and grinding [24], the Oka wear model is a modified version of the Archard wear model that takes into account the effect of particle size and hardness on the wear rate [25,26]. It was proposed in 2004 by Oka and Yoshida and has over time been used in oil and gas industrial applications. The Oka wear test involves sliding a pin against a flat surface under a specific load and speed. The wear rate is determined by measuring the weight loss of the pin [25]. The Archard wear model is a widely used model to predict the wear behavior of materials. The Archard wear model assumes that the wear rate is proportional to the load, sliding distance, and hardness of the material [27].

The *wear volume Q* in the Archard equation is expressed as [28]

(3) $$Q=WF_{n}d_{t}$$

where W is the *wear constant* of the material for Archard wear, $d_{t}$ is the *tangential distance*, and $F_{n}$ is the *normal force*.

On the other hand, the *wear volume per unit mass* $E_{\propto }$ for Oka wear is expressed as [25]

(4) $$E_{\propto }=65W^{-k_{1}}{\left(\frac{v}{104}\right)}^{{2.3H}_{v}^{0.038}}{\left(\frac{D}{0.326}\right)}^{0.19}$$

where v is the *velocity* of the particle, D is the particle *diameter*, and $H_{v}$ is the *Vicker’s hardness*.

Table 1 below shows some researchers that have applied Oka and Archard wear models with their limitations. This suggests that applications of wear models in the oil and gas environment could vary based on the target of the research; however, streamlined targets could result in limitations that hinder the full representation of real-world scenarios. The strain rate, surface finishing, composition, and environmental conditions are major variables that could influence the wear and shape memory effect (SME) and they are embedded in the Oka and Archer wear equations through the wear constant and geometry hardness. However, in this study, the focus is on the wear, therefore, the SM changes due to temperature were assumed not significant and were not considered. The Oka and Archard wear tests are known to predict wear behavior [24,25,29,30]; therefore, they were adopted in assessing the sand screen’s wear behavior during simulation. The *normal load*, *distance*, and *settling velocity* were all considered in simulation with time to see how they affect the wear rate as will be seen in the later sections of this paper.

## 2. Materials and Methods

### 2.1. Material Selection and Description

**Figure 2:**
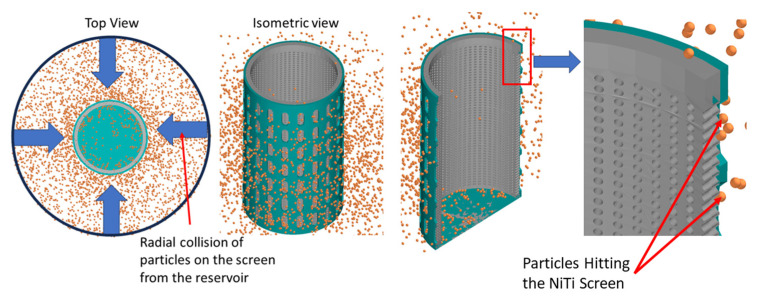
SMA sand screen geometry design and sand particle collision on a screen using Altair EDEM.

The material selected was NiTi and all key mechanical properties that contribute to its shape memory nature were considered, as shown in Table 2. Figure 2 is a representation of a standalone SMA sand screen experiencing sand particle collision on its surface, designed for the purpose of this study.

Together, *Poisson’s ratio (v), solid density (ρ), shear modulus (G)*, and *Young modulus (E)* offer a thorough insight into how a material will behave under various stresses and strains. They are used to design and test materials for several purposes in a variety of engineering and scientific applications. A measure of the elastic deformation of a material is known as *Poisson’s ratio (v).* It is dimensionless, provides insights into the elastic properties of a material, and represents the negative ratio of the transverse strain to the axial strain resulting from uniaxial stress. *Solid density* is another important property that describes the compactness of a material. It can reveal details about the material’s stiffness, weight, and heat and electricity conducting properties. Understanding how a material will react to bending or twisting depends on its shear modulus, which characterizes its resistance to shearing pressures. The *Young modulus* measures a material’s resistance to deformation under compression or tension and is significant in materials like metals and alloys where shear stresses can result in plastic deformation and failure. It offers details regarding the material’s capacity to support loads, withstand bending, and regain its original shape after deformation.

For Nitinol, the following parameters shown in Table 2 were recommended as mechanical characteristics.

**Table 2:** Mechanical properties of NiTi.

Mechanical Property	Review Ranges	Simulation Parameter	Reference
***Poisson’s Ratio (v)***	0.28–0.37	0.32	[37]
	0.32 longitudinal & 0.47 transverse		[38]
	<0.499		[39]
***Solid Density (ρ)***	6.45–6.6 g/cm^3^	6.5	[40,41,42]
***Shear Modulus (G)***	16–45 GPa	35	[43,44]
***Young Modulus (E)***	68 GPa	69	[45]
	70 GPa		[46]
	68.5 GPa		[40]

To comprehensively account for the geometry interaction with the sand particles, the study also incorporated the *coefficient of restitution (0.5)*, which quantifies the elasticity of colliding surfaces; *static friction (0.7)*, representing the friction necessary to initiate motion between stationary surfaces; and *rolling friction (0.15)*, which opposes the rolling motion of a surface. These considerations were essential to enhance the accuracy and completeness of the analysis.

### 2.2. Altair EDEM Simulation

**Figure 3:**
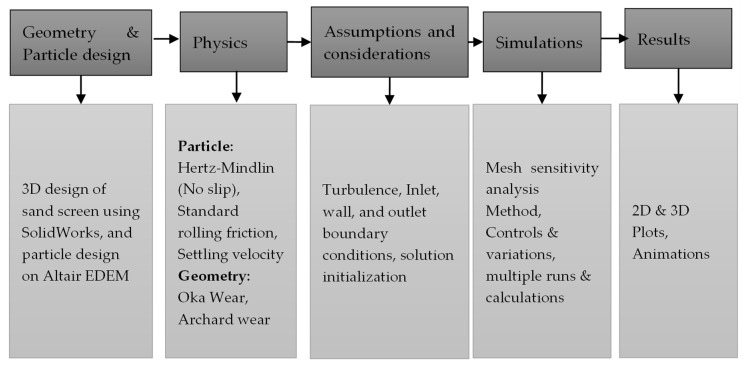
Flow of DEM simulation on SMA sand screen.

Figure 3 is the flow process during the DEM (discrete element method) simulation of Figure 2 on Altair EDEM. While the geometry of the sand screen and sand particle design and distribution were considered, the physics suggested the models used to describe the natural behavior of the sand particles as they collide with the walls of the sand screens. Hertz–Mindlin (no slip) and standard rolling friction were considered based on frictional behavior between contacting surfaces and two surfaces that are in relative motion due to rolling, respectively. Also, while the frictional coefficient of surfaces is relied on for Hertz–Mindlin, the sand particles are assumed to be free-flowing using standard rolling friction. This approach is particularly noteworthy as DEM is not commonly considered in describing geometry changes and continuous loading on tools, but the study demonstrates how it can be effectively utilized to improve the accuracy of the simulation [28].

***Settling the velocity of sand particles***

The ratio of sand production to gas production (in units of mass per volume) does not provide enough information to directly calculate sand velocity; therefore, additional information is needed, such as the particle size distribution and the fluid properties. This simulation does not consider the presence of gas; therefore, to get a representative value of the speed of sand particles, the study used an average settling velocity of sand particles in a gas well.

Knowing sand particles have a density of about 2650 kg/m^3^ (typical for quartz sand), the ratio of sand production to gas production can be used to calculate the sand velocity assuming a certain fluid flow rate. The continuity equation can be used to calculate the fluid velocity:

(5) Q=A×V

(6) $$A=\pi \times r^{2}$$

where *Q* is the *fluid flow rate*, *A* is the *cross-sectional area* of the wellbore, *r* is the *radius* of the wellbore, and *V* is the *fluid velocity*.

Using these values, fluid velocity can be solved with Equation (7):

(7) $$V=\frac{Q}{A}$$

Knowing the sand particle *diameter d*, *settling velocity* can be further estimated by first evaluating the *Reynolds number (Re)*, which in this case is turbulent for gas. According to [47], the movement of a spherical sand particle that is free-falling within a gaseous regime is governed by drag force, and Schiller and Naumann describe a *drag coefficient correlation (C_d_)* that best describes the process, as shown below, incorporating the Reynolds number.

(8) $$C_{d}=\frac{24}{Re}\left(1+0.15{Re}^{0.687}\right)+\frac{0.42}{1+25,000Re}$$

Furthermore, with the *drag coefficient correlation*, the *settling velocity* of the sand particles assumed to wear the surface of the screen can be estimated using the equation below [48]:

(9) $$V_{s}=\frac{4}{3}\frac{{(\rho }_{s}-\rho_{g})\times g\times d}{C_{d}\rho_{g}}$$

where *V_s_* is the settling velocity, $\rho_{s}$ is the density of the sand particles, $\rho_{g}$ is the density of the gaseous fluid, g is the acceleration due to gravity, and *d* is the diameter of the sand particles.

This calculation assumes that the sand particles settle in still fluid, which may not be the case in a flowing well. However, it gives an estimate of the settling velocity that can be used for comparison to the fluid velocity, which depicts reality as the fluid contacts the sandface. In this case, the settling velocity is much slower than the fluid velocity, suggesting that the sand particles would be carried along with the fluid flow and would not settle. However, in Altair EDEM, the sand velocity is calculated during particle tracking using the equation of particle motion as shown below, making it easier, as our simulations neglected the impact of gaseous fluids.

(10) $$x\left(t\right)=x_{0}+v_{0}t+\frac{1}{2}at^{2}$$

where *x(t)* is the particle position at time *t*, $x_{0}$ is the initial particle position, $v_{0}$ is the initial particle velocity, and a is the constant acceleration of the particle. 

A 3D representation of the sand particles colliding with the cylindrical surface of the sand screen (with circular perforations of 0.5 mm diameter) in a radial flow process during DEM simulation using Altair EDEM can be seen in Figure 2. 

## 3. Results and Discussion

### 3.1. Wear Analysis of SMAs

To understand the wear behavior of SMAs, numerical simulations using DEM were employed.

In a recent study, DEM simulations were used to model abrasive material loss in soil tillage via scratch tests [28]. It was found that the total wear volume was independent of the mesh size used in the simulation and was only dependent on the calculation time. This suggests that finer meshes do not necessarily lead to more accurate wear volume predictions, and coarser meshes can be used to reduce computational costs without compromising accuracy. However, the wear track characteristics were found to be dependent on the meshing of the geometry used, highlighting the importance of mesh quality in wear analysis. The load was identified as the dominant factor that determines the wear micro-mechanism and wear track characteristics in the simulations. This is consistent with previous studies on erosive wear of SMAs, which have shown that the wear rate increases with increasing load and abrasive particle size [14,49]. The wear behavior of SMAs can also be influenced by their microstructure, chemical composition, and surface properties [50,51].

To optimize the wear performance of SMAs under erosive conditions, it is essential to understand the underlying wear mechanisms and their dependence on the operating conditions. Numerical simulations using DEM can provide valuable insights into the wear behavior of SMAs and aid in the development of new materials with improved wear resistance, especially in sand screens for natural gas production.

### 3.2. Comparison of Wear Performance Using Oka and Archard on NiTi SMAs

According to Archard in 1953, the distribution and size of the total contact area, duration of contact, the shape of worn particles (layer removal, lump removal), and probability factor which demonstrates that not all contacts result in wear are necessary assumptions to consider the mechanical wear of surfaces [27]. 

**Figure 4:**
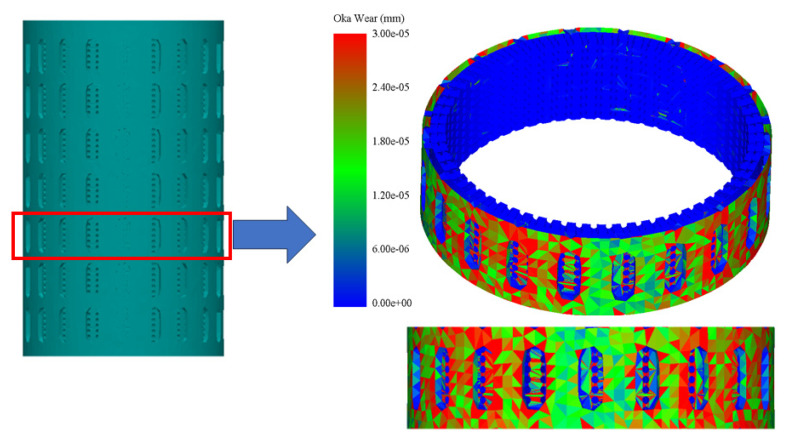
Sliced 3D view of Oka wear on NiTi SMA sand screen.

**Figure 5:**
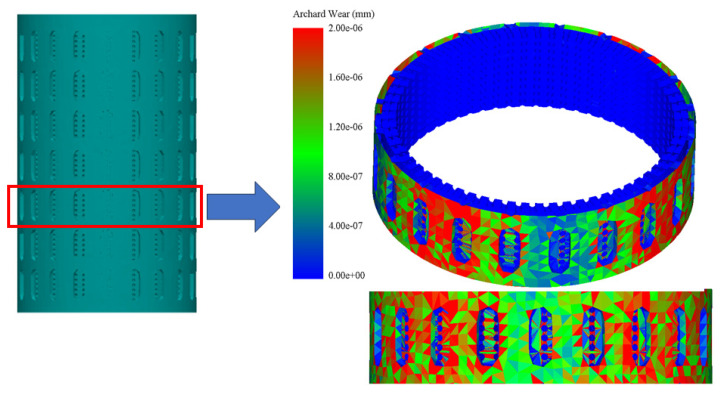
Sliced 3D view of Archard wear on NiTi SMA sand screen.

**Figure 6:**
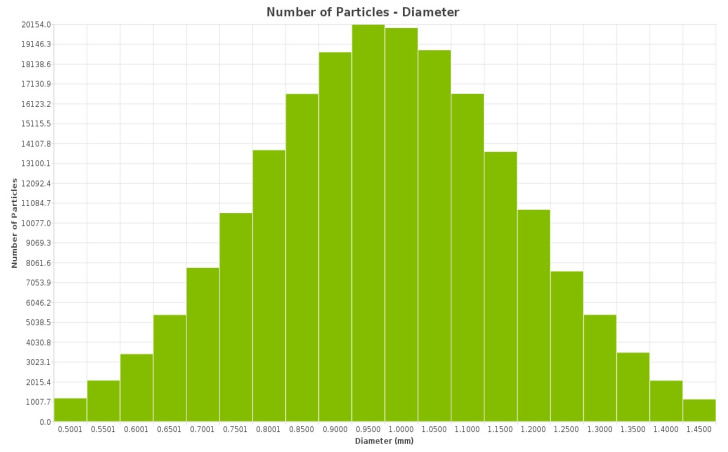
Particle distribution for mixed sand (0.5–1.5 mm).

**Figure 7:**
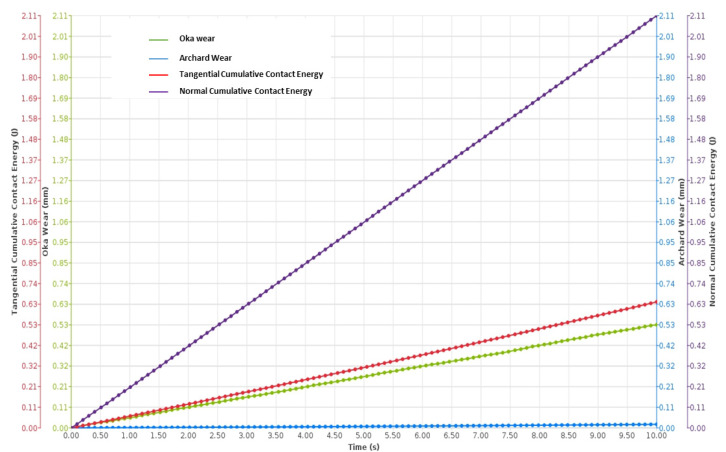
Wear plot of screen exposed to mixed sand particle distribution (0.5–1.5 mm).

**Figure 8:**
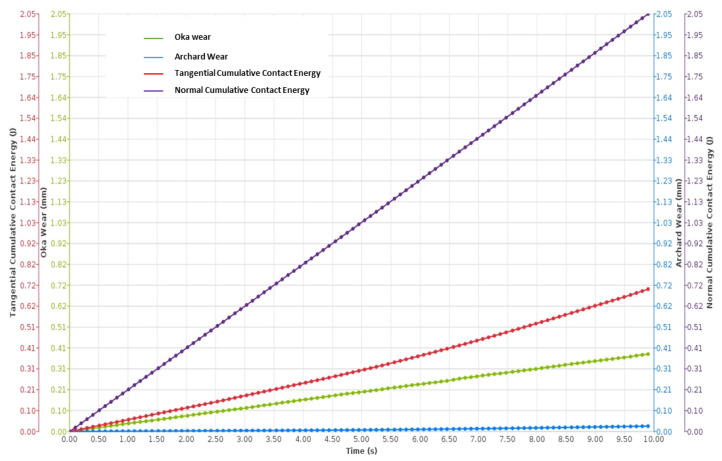
Wear plot of screen exposed to fixed fine sand particle distribution (1 mm).

The DEM simulation on NiTi, shown in Figure 4 and Figure 5, suggests that Oka wear is more relevant in analyzing the response of the sand screens to wear since it experiences higher wearing due to impact, while the Archard wear, which presents wearing due to abrasion, was not significant. The probability factor as suggested by [27] reflected on both the Archard and Oka wear on the screen as some parts of the screen were not affected by wear; however, this can be attributed to both the sand particle properties, distribution, and hardness, and the shape memory property of the geometry. The same wear response was compared based on the particle size distribution to understand the behavior of the material under fixed and mixed sand particle distribution. Figure 6 shows the distribution of the mixed sand particles used (0.5 mm to 1.5 mm), Figure 7 shows the plot for the mixed sand particles’ (0.5 mm to 1.5 mm) impact on the screen, while the plot in Figure 8 was for a fixed particle size of 1 mm.

The results of the wear response further revealed interesting findings regarding the influence of particle size distribution on wear behavior. Figure 7 demonstrated an increased wear effect when mixed grains (fine and coarse) were present, whereas Figure 8 indicated a lower wear effect with a fixed fine grain. These observations highlight the significance of particle size distribution in determining wear rates.

This can be attributed to the synergistic effect of different particle sizes causing more severe abrasion and wear damage on the surface or that a more uniform particle size distribution results in reduced abrasion and wear on the sand screen. 

The results from Figure 7 and Figure 8 provide support for the inference that in the scenario of sand screen wear during gas production, the Oka wear model is more representative than the Archard model. This conclusion is based on the comparison of the normal cumulative contact energy to the tangential cumulative contact energy in both figures, which relates to the drag coefficient discussed earlier. The dominance of the normal cumulative contact energy over the tangential cumulative contact energy implies that the wear mechanism in this case is predominantly driven by impact rather than abrasion. This suggests that the particles impinging on the Nitinol sand screen during gas production cause more significant wear through high-energy impacts rather than gradual abrasion due to tangential forces. Also, supporting this, the tangential energy in Figure 7 and Figure 8 suggested a higher effect from fixed fine particles than coarse ones.

Overall, outcomes of the DEM simulation support earlier reviews from Table 1 that suggested that wear models are better understood when the material, mechanism, and particle activities are collectively investigated.

### 3.3. SMA Microstructural Nature of Nitinol Influencing Wear Resistance

The microstructure of Nitinol is primarily composed of nickel and titanium, which play a crucial role in its unique mechanical properties and resistance to wear. Nitinol typically exhibits a two-phase microstructure consisting of a matrix of B2 austenite and a B19 martensitic phase [52].

In the annealed state, Nitinol has a fully austenitic microstructure, which provides it with excellent ductility and formability [53]. When Nitinol is subjected to a temperature change or mechanical deformation, it undergoes a reversible phase transformation from austenite to martensite and vice versa, known as the SME [52]. This phase transformation gives Nitinol its remarkable superelasticity and shape memory properties.

It is safe to infer that Nitinol’s microstructure contributes to its favorable mechanical properties to wear. The presence of the martensitic phase enhances its hardness and strength, in combination with the self-healing effect, making it more resistant to plastic deformation and wear [52,54].

Traditionally, steel has been used in fabricating sand screens due to its strength, availability, and cost [55,56,57]. However, in comparison to Nitinol’s wear resistance, there are several factors that contribute to Nitinol’s superior performance in certain wear scenarios, and these could be its ductility and flexibility, its corrosion resistance, low friction coefficient, and self-lubricating effect of its thin oxide layer. However, it is important to note that the wear resistance of Nitinol can still vary depending on specific wear conditions, such as load, contact area, velocity, and the nature of its surface and particles in contact. Therefore, it is essential to consider the specific application and wear parameters when evaluating the relative wear resistance of Nitinol and steel.

The grain boundaries could also play a crucial role in the wear resistance of Nitinol. High-angle grain boundaries hinder dislocation movement, impeding wear deformation [58,59]. Furthermore, grain boundary sliding and grain refinement can enhance wear resistance [60]. Also, the formation of intermetallic compounds at grain boundaries or within the microstructure of Nitinol can affect its wear resistance. Studies have shown that intermetallic phases, such as Ni4Ti3 and Ni3Ti, contribute to the material’s hardness and resistance to wear [61,62]. Since these studies have provided evidence of the impact of grain boundaries and the intermetallic phases on wear behavior, it can be inferred that the characteristics that give nitinol its shape memory behavior also position it as a wear-resistant material. The Hall Petch effect that suggests the presence of smaller grains influencing hardness [61] also supports this inference. Also, a stable microstructure ensures that the material maintains its wear-resistant properties over extended periods [63,64]. Therefore, the stability of Nitinol’s microstructure, including its phase composition and crystal structure, can also contribute to its wear resistance.

In comparison to traditional materials as investigated by Liu and Li in 2001 using Archard wear in Table 3, materials exhibit wear loss when in contact with an external load. However, it has been observed that solely ranking materials based on their hardness may not always be adequate because of the complexities of their microstructure. This is primarily because the *wear coefficient*, denoted as K in Archard’s equation, tends to vary among different materials [65]. Materials that possess high elasticity or pseudoelasticity pose a challenge for Archard’s equation as seen in Table 3, as it fails to provide reasonable predictions consistent with experimental observations.

**Figure 9:**
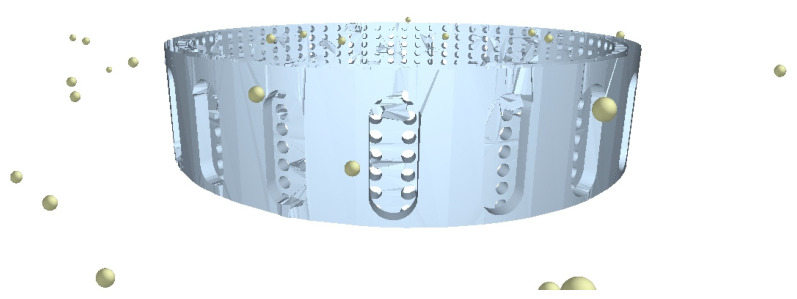
Ridges and plugging formed during wear loss of SMA sand screen at high settling velocity using Altair EDEM.

Finally, in the plastically formed ridges during geometrical change as seen in Figure 9, both force and distance influence the nature of the plane in front of the particle. This was possible due to the addition of a deformation option during the DEM simulation, suggesting an elastic–plastic behavior of the material when impacted upon by the sand particles [66]. The 3D visualization of the simulation displayed short drags, indicating the formation of plastically deformed ridges on the surface of Nitinol. This finding further supports Nitinol’s wear-resistant nature.

Figure 9, as obtained from the Altair EDEM simulation, indicates that Nitinol exhibits promising wear resistance when used as sand screens and analyzed using the Oka wear model, which is more representative of wear action at the sandface. The minimal wear observed suggests that Nitinol’s mechanical properties and microstructure contribute to its ability to withstand erosive forces. The formation of short drags and plastically deformed ridges at high settling velocities further supports the wear-resistant nature of Nitinol. However, it is important to note that Altair EDEM does not include a shape memory model, which limits the simulation’s ability to evaluate the SME of Nitinol and a laboratory analysis will be needed to observe the response post-wear.

### 3.4. Validation of the Numerical Simulation with Experimental Findings

The numerical simulation compared the Oka and Archard wear models on a NiTi sand screen to suggest the model that exhibits superior relevance for the mechanical analysis of such systems. To further elaborate, reviews from the earlier sections suggest that the Oka wear model is more significant when considering impact (indentation), while Archard’s significance is on abrasion (scratch). However, the concurrent implementation of these wear models in sand screen wear simulations to ascertain their respective applicability remains notably absent. The simulation further identified both wear model effects on the screen, but Oka proved more significant. Though there is no specific research that extensively covers sand impact wear on NiTi used for screening, this section attempts to justify the simulated result using experimental analysis of both scenarios considering morphological responses, wear depth, and the suitability of NiTi as suggested by simulation for screens over traditional screen metals.

**Figure 10:**
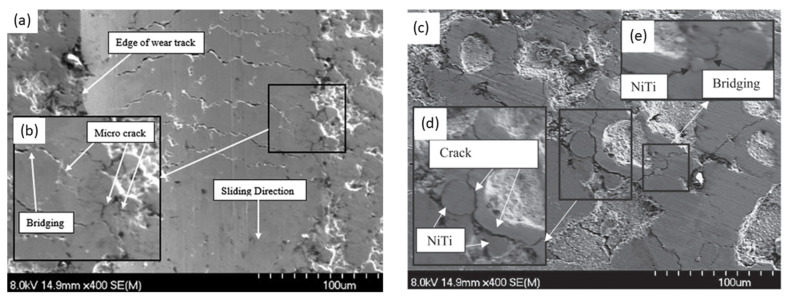
Surface morphology of wear (**a**) due to impact showing edges of wear tracks and cracks (**b**) wear due to impact showing microcracks and bridging (**c**) on a 2 g NiTi coating showing crack and very mild drag (**d**) extended cracks due to impact (**e**) bridging effect [67].

**Figure 11:**
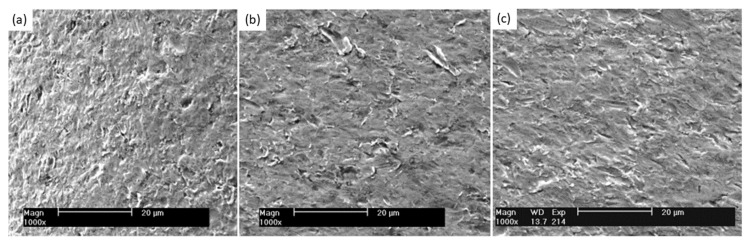
Surface morphology of wear due to impact on NiTi at 90° (**a**), 45° (**b**), and 20° (**c**), showing both indentations and drags [68].

Figure 10 suggests that impact wear, which is observed using the Oka wear model, presents localized surface damage without extensive drag; however, that of abrasion, which is measured using Archard wear, depicting the surface removal due to scratch presents extensive drag on the surface. The study’s [67] investigation of NiTi’s performance in the context of scratch and indentation wear reveals a noteworthy observation. This observation corroborates the earlier simulation outcomes, which indicate high Oka wear and low Archard wear on the sand screen. Also, under the impact, radial crack lengths (as seen in “c” and “d”) are more prominent than drags [67], which support the propagation of deeper and localized wear on the screen surface. Figure 10 and Figure 11 further buttress the fact that while traces of drag effects may persist within the context of an impact wear test, their manifestation remains considerably subdued in comparison to the pronounced occurrence of localized wear phenomena.

Figure 11 showed a combined appearance of both indentation and drag in an impact test on NiTi. According to [68], the surface wear behavior can be influenced by the angle of impact, as seen in Figure 11, the indentations are seen more at 90° than 20°, while scratch is otherwise, which aligns with what happens to screens at the sandface at 90° radial impact. This can also be validated by the study that confirms NiTi as an anisotropic intermetallic compound that responds to particle impact at different angles [69]. The relationship between indentation force and elastic modulus has been examined and demonstrated to be directly proportional [70]. This observation is consistent with the outcomes of the present investigation, which indicate that the Oka wear model, which focuses on impact-related factors, more accurately represents the significant wear losses experienced during impact. Fang, et al.’s study on wear on pipelines further supports this as it demonstrated the Oka wear model to agree with simulated results of the impact of solid particles, while the Archard wear model was adopted when locating the area of maximum wear due to observed concentrated scratches on the pipe [71]. This underscores the distinct nature of wear mechanisms under impact and abrasion conditions, thereby contributing to a more comprehensive understanding of the intricate interactions between wear models and sand screen wear dynamics.

Since the focus is more on the surface material response to wear and not the overall screen orientation, traditional materials used in screens that have been applied in different facilities experiencing impact wear will be compared with the NiTi simulated results of this study at similar erosion conditions.

Carbon steel undergoing impact wear by particles of 0.05 mm at flow velocities of 0.5 m/s, 1 m/s, and 2 m/s were compared with simulated NiTi results at the same conditions, as shown in the figure below.

**Figure 12:**
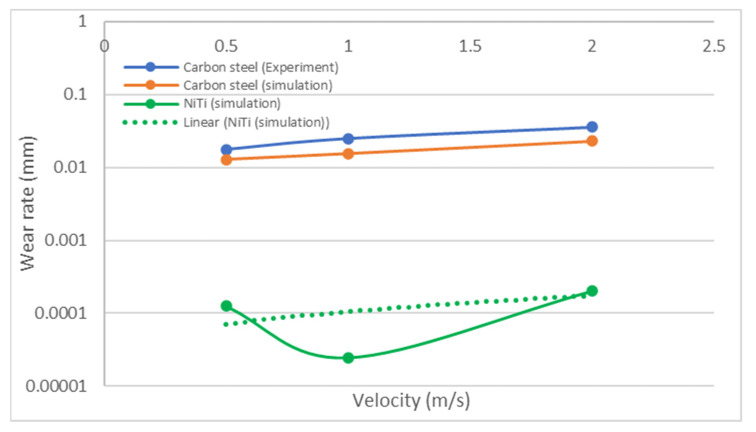
*Wear rate* to *impact velocity* at 0.5 m/s, 1 m/s, and 2 m/s from a 0.05 mm *particle size* on carbon steel (redrawn from [72]) in comparison to NiTi from this study.

**Figure 13:**
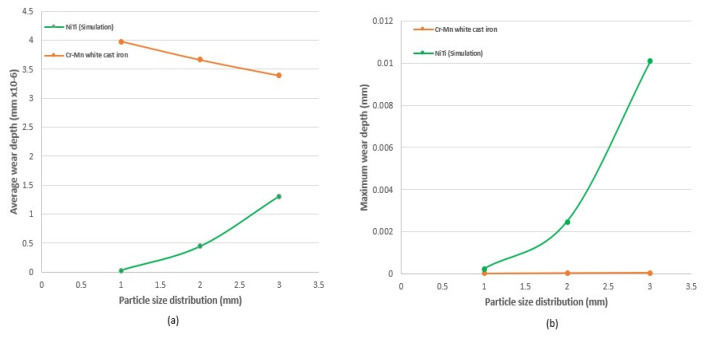
Plot of (**a**) *average wear depth* and (**b**) *maximum* wear depth, against *particle size* (diameter) of Cr–Mn white cast iron (redrawn from [73]) in comparison to the simulated result of NiTi for this study at 1 mm, 2 mm, and 3 mm using Oka wear.

Figure 12 shows a comparison of carbon steel and NiTi; on the wear rate axis, a logarithmic scale was used to show the exponential differences between both wear rates. This suggested that the wear on surfaces of carbon steel is more significant when compared to that of NiTi and increases as the impact velocity increases. Another result (Figure 13) was derived from a research investigation into the wear characteristics of a centrifugal pump under impact wear from sand particles. The data revealed that the average wear depth observed on Cr–Mn white cast iron blades was more than that observed on the NiTi material utilized in this study, which also experienced impact wear under sand particles of the same size distribution. However, there were slight differences as the particle size increased. In the case of NiTi, it has been observed that the average wear depth tends to increase with an increase in particle size. However, with Cr–Mn white cast iron centrifugal pump blades, the wear depth exhibits a reduction as the particle size increases. It can be inferred that the observed changes in the surfaces of the Cr–Mn white blade and NiTi screen may be attributed to the occurrence of dynamic rotatory operation in the former and static operation in the latter. However, both surfaces were subjected to impact stress, as shown by the Oka wear model. In contrast, the maximum wear depth for NiTi was increasing at a higher rate with increasing particle diameter than the Cr–Mn with cast iron. These comparisons agree with the simulated results of Figure 7 and Figure 8, noting that the wear depth during wear increases as particle diameter increases. Recall from simulated results of Figure 7 and Figure 8 that fixed fine particle size with higher cumulative contact energy had a lower wear rate compared to mixed (which is a combination of fine and coarse), meaning the prevalence of larger particle sizes in the mixed configuration led to an elevated wear potential. Given the focal emphasis on wear depth within this comparative assessment, the alignment with the principles of the Oka wear model becomes particularly conspicuous.

**Figure 14:**
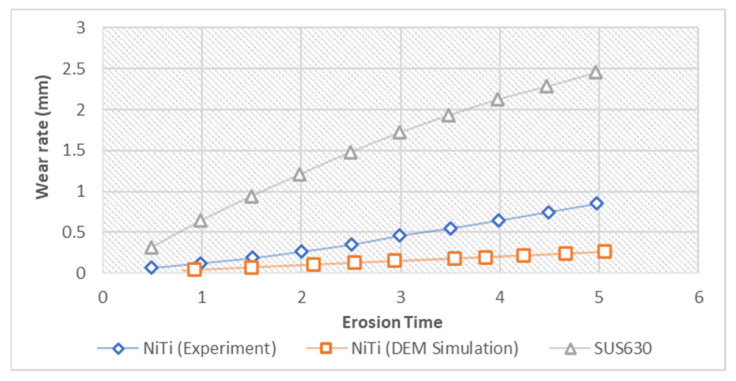
*Wear rate* plot of NiTi and SUS630 at an *impact angle* of 30° (redrawn from [74]) compared to DEM simulation from this study using the Oka wear model.

To justify the effectiveness of NiTi over steel, which is traditionally used for sand screens, SUS630 was compared with NiTi under 0.3MPa as shown in Figure 14 and the results suggested that NiTi experiences a lower wear rate than SUS630. From the plot, both the results of NiTi wear during experimental analysis and that of this study’s simulation were much lower than that of SUS630 wear. This can also be corroborated with findings in Table 3, which suggest low wear when NiTi was used compared to stainless steel or Al alloy [65].

The study’s [74] microstructural analysis also opined that the hardness of NiTi at 90° and 45° increases (381 HV and 405 HV) more than that of SUS630 (311 HV and 334 HV) post-wear. The difference in hardness provides compelling evidence that the hardness of the eroded surface in NiTi surpasses that of SUS630, consequently establishing a plausible link to heightened drag forces and augmenting wear resistance. Lastly, a study that compared TiC–stainless steel and TiC–NiTi blend also corroborated that the NiTi blend revealed better wear resistance of 0.4 mg/min over the TiC–stainless steel of 4.0 mg/min [75].

## 4. Conclusions and Recommendations

### 4.1. Summary of Key Findings, Significance, and Contributions of the Study

The present study posits the significance of the Oka wear model as a more pertinent consideration in the investigation of sand screen wear. This assertion is based on the observation of considerably higher wear values as seen on the plots and deformations seen on the 3D views associated with Oka wear when compared to those attributed to Archard wear, which is found to be negligible in this context and validatory information from similar experimental analysis of Oka and Archard wear models. The rationale behind the prominence of the Oka model lies in its emphasis on impact as the predominant wear mechanism, as opposed to abrasion. Such a focus aligns with the activities occurring at the sandface, thereby rendering the Oka model more representative of the wear phenomena encountered in this domain.

The findings from reviews and application in simulation also place NiTi as a superior material for wear resistance when compared to traditional screening materials. Furthermore, the microstructural nature of NiTi, including the presence of austenite and martensite phases, grain boundaries, intermetallic compounds, drag response, and the SME, influences its wear resistance. Understanding these microstructural elements, bonds, and behaviors is vital for optimizing NiTi’s wear resistance properties. Further investigations should focus on characterizing the microstructural changes post-wear, the effect of temperature, and their influence on Nitinol’s wear behavior.

### 4.2. Limitations and Future Research Directions

One limitation of the present study is the inability to observe the SME within the DEM simulation due to the inherent constraints of the Altair EDEM software (v2022.2). Nonetheless, the investigation focused on exploring the mechanical properties of Nitinol, a well-established SMA, as identified through literature reviews. The primary objective was to determine if these SMEs play a role in influencing the material’s mechanical responses when employed as sand screens and subjected to wear. Consequently, future investigations could potentially capitalize on unveiling the SME post-wear by employing laboratory setups supplemented with computed tomography (CT) scans to analyze the surface structural deformation and possible recovery post-wear.

## Figures and Tables

**Table 1:** Applications of Oka and Archard wear models.

Wear Model	Focus	Limitation	Ref.
Oka wear	The influence of particle impact angle, particle velocity, and particle size distribution on wear in a pipe bend.	The study adopted Oka, McLaury, Finnie, and Generic wear models in its investigation of a pipe bend, suggesting that researchers are constantly seeking models’ best fit for specific wear scenarios. However, the clarification regarding whether the wear observed at the extrados of the pipe near the bend exit was primarily influenced by impact and abrasion was not explicitly addressed. Variation in particulate sizes of spherical-shaped sand particles in the discrete phase model was rather primary, potentially necessitating a critical review of the wear mechanisms for a more comprehensive understanding of wear phenomena in pipe bends.	[31]
	To investigate the effect of particle size on wear in turbulent slurry flow using empirical (Oka and Huang) and analytical (Cheng) wear models.	Oka, Huang, and Cheng wear models were all used in the inverse analysis study despite their similar impact-driven wear nature. The scenario involved rotation which might have tilted towards abrasion influence, but models like Archard were not considered. Though results gave irregular impact velocities, the study still suggested the Oka wear model as the most reliable in performance prediction. Other limitations identified in the study were reference speed and removal of plastic damage.	[32]
	The focus was on the effect of surface roughness on the velocity, pressure, and particle trajectories within a cyclone.	An increase in wall roughness resulted in a decreasing wear rate using the Oka wear model in this case. The nature of the material used for the cyclone was not specified or compared to help the researcher opine whether there are microstructural characteristics necessary for the resistance to wear as roughness increases. Particle size influence was less significant. Unlike this [33], in the later part of this paper, the present study characterized the SMA sample to reflect the response due to wear.	[33]
Archard wear	The paper compares the critical plane-based method and the deviatoric strain amplitude-based method for evaluating fretting fatigue life and corrects for surface wear damage using the Archard wear model.	The limitation of the paper with respect to Archard wear is that the authors assume a linear relationship between wear volume and time, which may not hold for all materials, contact energies, and wear conditions. The paper does not consider the effect of wear debris accumulation and the potential for third-body abrasive wear, which can significantly affect the fretting fatigue behavior of a material.	[34]
	The paper uses numerical analysis to simulate the wear behavior of a die and applies the Archard wear model to predict the wear volume and wear rate of the die.	The limitation of this research was that the authors assumed a constant wear coefficient for the titanium alloy material throughout the simulations, which may not accurately reflect the wear behavior of the material under different conditions. The paper does not consider the effect of adhesive wear or abrasive wear, which can also contribute to the wear of the die. Finally, the study is focused on a specific application and material, so the findings may not be directly applicable to other materials or applications.	[35]
	The paper uses a combination of finite element analysis (FEA) and multi-body dynamics (MBD) to simulate the contact and wear behavior of the cervical total disc arthroplasty and applies the Archard wear model to predict the wear volume and wear rate of the ultrahigh-molecular-weight polyethylene insert.	The authors assume a constant wear coefficient for the UHMWPE material throughout the simulations, which may not accurately reflect the wear behavior of the material under different conditions. Wear predictions were made based on sliding distance and contact stresses as unique to the Archard model. A comparison of other wear model responses would have been more informative for the disc prosthesis being studied. This study clarified that the wear models are not limited to metals or alloys, making it evident to continuously investigate unique responses of materials to wear.	[36]

**Table 3:** Comparison of experimental and theoretical wear losses [65].

Materials	Wear Loss in an Experiment (mm)			Wear Loss in Theory (mm)		
	Low Load	Mid Load	High Load	Low Load	Mid Load	High Load
Stainless steel	12.00	19.00	29.00	11.95	19.46	27.15
Al alloy	19.00	30.00	42.00	23.66	34.51	43.67
NiTi	10.00	14.00	21.00	15.04	20.97	28.84

## Data Availability

Data are contained within the article.
